# What matters: non-pharmaceutical interventions for COVID-19 in Europe

**DOI:** 10.1186/s13756-021-01039-x

**Published:** 2022-01-09

**Authors:** Yan Liu, Qiuyan Yu, Haoyu Wen, Fang Shi, Fang Wang, Yudi Zhao, Qiumian Hong, Chuanhua Yu

**Affiliations:** 1 Department of Epidemiology and Biostatistics, School of Public Health, Wuhan University, Hubei, China; 2 Department of Epidemiology and Medicine Statistics,Public Health and Management School, Wenzhou Medical University, Zhejiang, China; 3 School of Public Health, Xuzhou Medical University, Jiangsu, China

**Keywords:** Non-pharmaceutical interventions, COVID-19, Europe, Lasso regression, Quantile regression

## Abstract

**Objectives:**

The purpose of this study is to describe the situation of COVID-19 in European countries and to identify important factors related to prevention and control.

**Methods:**

We obtained data from World Health Statistics 2020 and the Institute for Health Metrics and Evaluation (IHME). We calculated the Rt values of 51 countries in Europe under different prevention and control measures. We used lasso regression to screen factors associated with morbidity and mortality. For the selected variables, we used quantile regression to analyse the relevant influencing factors in countries with different levels of morbidity or mortality.

**Results:**

The government has a great influence on the change in Rt value through prevention and control measures. The most important factors for personal and group prevention and control are the mobility index, testing, the closure of educational facilities, restrictions on large-scale gatherings, and commercial restrictions. The number of ICU beds and doctors in medical resources are also key factors. Basic sanitation facilities, such as the proportion of safe drinking water, also have an impact on the COVID-19 epidemic.

**Conclusions:**

We described the current status of COVID-19 in European countries. Our findings demonstrated key factors in individual and group prevention measures.

## Background

Since 7 December 2020, there have been 66,243,918 cumulative cases and 1,528,984 cumulative deaths worldwide according to data from the World Health Organization (WHO) on coronavirus disease (COVID-19). The WHO declared COVID-19 to be a pandemic on 12 March 2020; a pandemic is a global public health emergency of high international concern. COVID-19 is an acute respiratory infectious disease transmitted mainly through aerosols and droplets [1], and is characterised by higher infectivity but lower mortality than SARS, so we should strengthen its prevention and control [2].

The chief non-pharmacological prevention and control measures included individual and group prevention. Personal prevention primarily included wearing masks, washing one’s hands, proper social distancing, and reducing participation in outdoor activities. Group prevention mostly encompassed travel restrictions, home isolation, the closure of educational facilities, prohibitions on public gatherings, all commercial activities, and non-essential commercial activities. The effect of prevention and control may also be related to medical resources (e.g. doctors, nurses, pharmacists, ventilators, ordinary beds, ICU beds), as well as the country’s economic situation and ability to respond to public health incidents (e.g. gross domestic product [GDP] and global health security [GHS]).

Switzerland regards testing as the central method to control COVID-19 [3]. A meta-analysis showed that medical masks and N95 masks can protect against viral respiratory infection [4]. Community-wide mask wearing can control COVID-19 by preventing contact with large amounts of saliva and respiratory droplets [5]. Hand hygiene is the most important factor in control activities and infection prevention [6]. The use of antiseptic hand soaps leads to a greater reduction in the number of microorganisms compared with regular soaps [7]. However, the toxicity and stability of surface disinfectants are issues that should be further investigated [8]. The findings of a systematic review and meta-analysis support the idea that physical distancing of 1 m, wearing face masks, and eye protection prevent the person-to-person transmission of COVID-19 [9].

The first country in Europe where the COVID-19 outbreak occurred was Italy. On 9 March 2020, the Italian government implemented a comprehensive ‘blockade’ policy, mostly consisting of travel restrictions, mandatory stay-at-home orders (except for health problems, emergencies, or regulated shopping for necessities), and the temporary closure of non-essential businesses and shops, which lasted until 3 May 2020. In the following months, many European states adopted similar measures [10]. In March 2020, 47 countries did not allow commercial flights to land [11]. Moreover, it became necessary to develop an international framework to outline the method, time, and scale of travel restrictions according to the stage of the epidemic [12]. A cross-sectional study suggests that issuing a stay-at-home ban may help limit the spread of COVID-19 cases [13]. On 12 March, Norway issued stricter measures and instituted quarantines for those who entered the country. That same day, the government closed all schools and kindergartens; training centres and offices for psychologists and physiotherapists; hair salons; and swimming pools, and forbade cultural and sporting events. Sweden chose a different strategy: Kindergartens, elementary schools, other businesses, and training facilities remained open, and children’s sports continued [14]. Many sporting events were restricted or cancelled to limit the spread of disease [15]. Some non-essential businesses, such as vape shops, were closed [16].

The disease incidence and mortality of COVID-19 were related to country healthcare resources and economic status [17]. In Italy, 16% of hospitalised COVID-19 patients required intensive care. Compared with Germany, the medical resources in northern Italy became overwhelmed by the increase in patients. In contrast, Germany had a wider distribution of cases and was able to make better use of its resources. The high mortality rate in Italy may reflect the relationship between the availability of medical resources and outcomes [18]. In addition, the prevention and control effects of COVID-19 may be tied to indicators such as GDP and GHS [19].

## Materials and methods

### Research objective and data sources

We obtained data from World Health Statistics 2020 and the Institute for Health Metrics and Evaluation (IHME). The IHME is an independent population health research centre at the University of Washington Medicine. We selected 51 countries in Europe as our research objective. There are 53 countries in Europe according to WHO regional groupings. The data from Monaco and Turkmenistan were incomplete, so we discarded those two countries.

Where does the IHME obtain its data? These forecasts include data from local and national governments, hospital networks and associations, the WHO, third-party aggregators, and a range of other sources.

For testing data, the IHME relies primarily on data reported by Our World in Data. However, Cyprus, Italy, and Spain used government data. The IHME obtains hospital resource data from sources such as government websites, hospital associations, the Organisation for Economic Co-operation and Development (OECD), the WHO, and published studies. For population density, they used gridded population count estimates for 2020 at the 1 × 1 km (km) level from World-Pop. For mobility index data, the IHME used anonymised, aggregated data from Google, Facebook, and Apple. Their data on mask use come from the Premise, Facebook Global Symptom Survey (research based on survey results from the University of Maryland Social Data Science Center), the Kaiser Family Foundation (KFF), and the YouGov COVID-19 Behaviour Tracker survey.

### Statistical analysis method

#### Changes in Rt under different prevention and control measures

For this study, we used the time-dependent basic reproduction number method. The time-dependent reproduction number (Rt) is the average number of secondary cases of a single infected person during t day of infection. Rt is usually applied to describe the transmission characteristics of pathogens during an epidemic, and can also evaluate the effectiveness of interventions. When Rt is greater than 1, it indicates that the number of infections is rising rapidly; when Rt is less than 1 and close to 0, it suggests that the epidemic has been effectively controlled [20].

### Lasso regression

Traditional linear regression belongs to the subset selection method, and the chief approach for screening variables is the ordinary least squares (OLS) technique. However, OLS has some defects: Variable selection is separated from model parameter estimation, so the model error increases; small changes in variables have a great influence on variable selection. Variable selection is not suitable for high-dimensional data [21, 22]. The least absolute shrinkage and selection operator regression (lasso regression) is the representative regularisation method that can effectively optimise the OLS estimation and treatment overfitting problem [23]. By introducing a penalty term into model estimation, lasso regression can obtain higher prediction accuracy and model generalisation ability. It can also effectively address overfitting and multicollinearity problems [24–26]. The specific formula can be expressed as:

$$\hat{\beta }\left( {lasso} \right) = \arg \mathop {\min }\limits_{\beta } ||y - \sum\limits_{j = 1}^{p} {X_{j} \beta_{j} ||^{2} } + \lambda \sum\limits_{j = 1}^{p} {|\beta_{j} } |$$

The first part represents the standard OLS loss function, while the second part denotes the penalty function. It represents the tuning parameter for controlling the degree of regression coefficient compression. When λ is ≥ 0, the greater the value, the stronger the penalty. When λ = 0, the loss function does not penalise the model. Lasso regression shrinks some coefficients and sets others to 0, and tries to retain the good features of both subset selection and ridge regression [27]. Researchers adopt different forms of penalty functions according to the different characteristics of independent variables in regression analysis on the basis of lasso regression. Many kinds of regularisation models have been established and developed, such as relaxed lasso [28], adaptive lasso [26], Bayesian lasso [29], fused lasso [24], group lasso [30], and elastic net [25].

### Quantile regression

Quantile regression (QR) estimates the linear relationship between the different quantiles of the dependent variable and the independent variable. Both QR and ordinary least squares (OLS) can solve specific minimisation problems. The estimation of OLS regression is grounded in the smallest residual square, and the estimation of QR is rooted in the smallest weighted absolute value residual. The minimum weighted absolute deviation of quantile regression is as follows:

$$\min \left\{ {w_{t} |y_{t} - \alpha |} \right\} = - \sum\limits_{{i:y_{i} < \alpha }}^{T} {\left( {1 - \tau } \right)} \left( {y_{t} - \alpha } \right) + \sum\limits_{{i:y_{i} \ge \alpha }}^{T} \tau \left( {y_{t} - \alpha } \right)$$

The purpose of quantile regression is to calculate the regression coefficients of different quantile values of the dependent variable. It can comprehensively display all data information to a certain extent. Thus, it has unique advantages over traditional linear regression models. Especially for the condition of a non-normal distribution, quantile regression is more comprehensive and accurate than traditional linear regression coefficient estimation [31]. We used R software 4.0.2 and Stata 15.0 for all of our statistical analyses.

## Results

### Changes in Rt under different prevention and control measures

We plotted the changes in Rt values of 51 countries in Europe. After an outbreak period ranging from 2–3 months, the Rt of France, Denmark, Belgium, Armenia, Germany, the Netherlands, Spain, Portugal, Moldova, Sweden, Tajikistan, Turkey, and Uzbekistan were basically stable, and the value fluctuated at approximately 1. Russia and Ukraine had a longer outbreak period, but the Rt afterwards also stabilised at approximately 1. The Rt of other countries fluctuated to varying degrees. The general trend of changes in Rt in most European countries showed an increasing trend after October and exceeded 1.

**Fig. 1:**
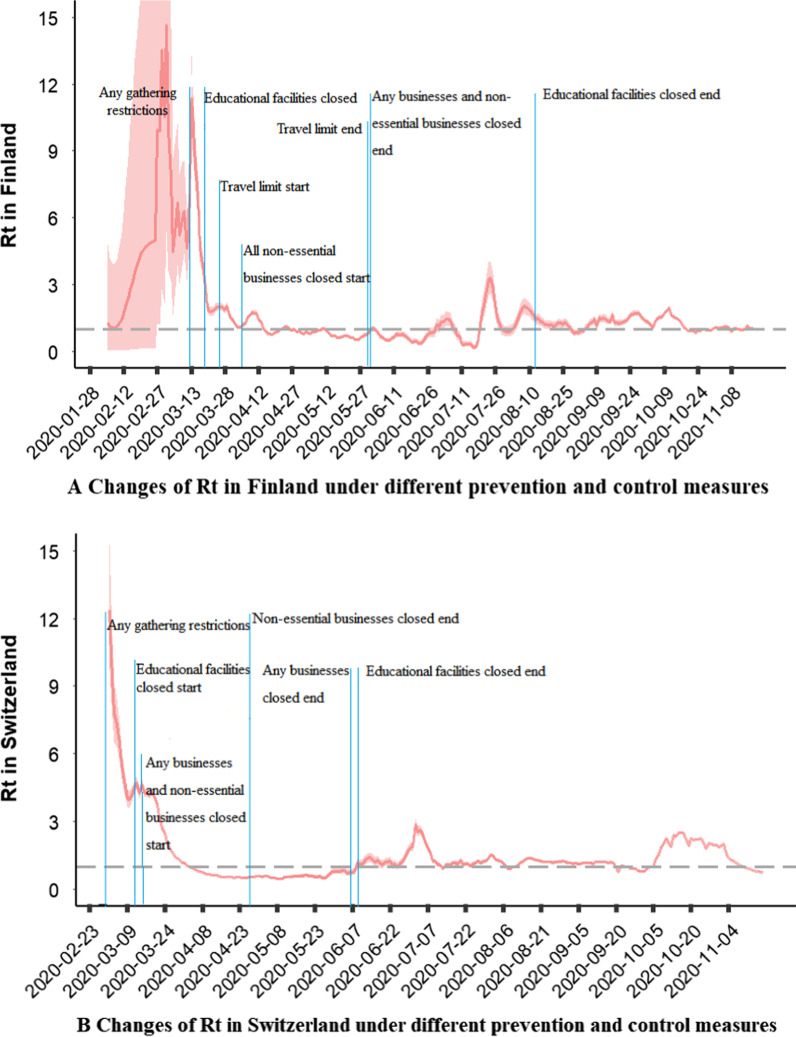
**a** Changes of Rt in Finland under different prevention and control measures **b** Changes of Rt in Switzerland under different prevention and control measures

Let us take Finland and Switzerland as examples. In Fig. 1a, we describe the changes in the Rt value in Finland under different prevention and control measures. The first case of COVID-19 occurred on 28 January in Finland, and on 18 March, any business activities were banned and educational facilities were closed. Travel restrictions were implemented on 25 March, and unnecessary business activities were prohibited on 4 April. Prevention and control effects were achieved, and the Rt value dropped below 1 under the above four powerful group interventions. On 29 May, the government ended travel restrictions. On 1 June, the government ended the ban on restricting any business activities and non-essential commercial activities. Due to the relaxation of the intervention policy, the Rt value rebounded and rose. On 24 July, the Rt value reached a small peak of 3.296. On 13 August, when the Rt value dropped to 1.398, the restriction on school facility closure ended. Then, on 11 October, the Rt value fluctuated repeatedly, and on 11 October, it reached 1.966.

In Fig. 1b, we describe the changes in Rt values in Switzerland under different prevention and control measures. Cases first appeared in Switzerland on 23 February, and large gatherings were banned on 28 February. On 13 March, education facilities were closed. On 16 March, any commercial activities and non-essential commercial activities were prohibited. Under the above group intervention, a certain prevention and control effect was achieved, and the Rt value dropped below 1. Thus, on 27 April, the government ended the restrictions on non-essential commercial activities. On 6 June, the government ended the ban on any commercial activities. On 8 June, the government decided to end the closure of school education facilities. Due to the relaxation of the intervention policy, the Rt value rebounded and rose. On 2 July, the Rt value exhibited a small peak of 2.88. Subsequently, the Rt value dropped to approximately 1. On 16 October, there was a small peak of 2.51. The graphs of Rt changes in other countries can be seen in the supplemental materials.

### Results of lasso regression

In this study, we explored the relationship between two dependent variables and 17 independent variables, including cumulative morbidity (Y1) and cumulative mortality (Y2). The independent variables were the density of medical doctors/per 10,000 people (X1), the density of medical nursing and midwifery personnel/per 10,000 people (X2), the density of medical pharmacists/per 10,000 people (X3), GDP per capita (US dollars per capita) (X4), the proportion of the population using safely managed drinking-water services (X5), total tests (X6), the mask use rate (X7), the mobility composite (X8), excess bed capacity for COVID-19 (X9), the ICU excess bed capacity for COVID-19 (X10), travel restrictions (X11), stay-at-home orders (X12), the closure of educational facilities (X13), restrictions on gathering (X14), business closures (X15), non-essential businesses being ordered to close (X16), and the GHS index (X17).

**Fig. 2:**
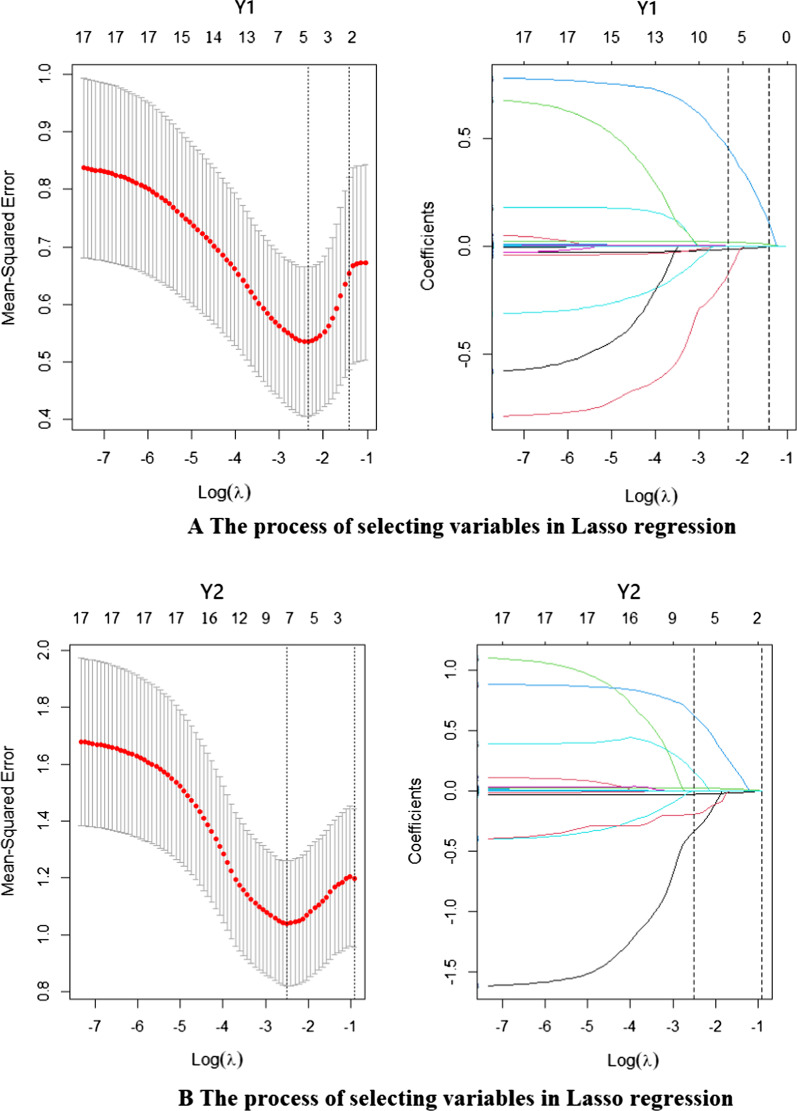
**a** The process of selecting variables in Lasso regression and **b** the process of selection variables in Lasso regression

We finally screened five out of 17 variables related to cumulative morbidity (Y1) and seven variables related to cumulative mortality (Y2) after lasso regression selection. The specific content is shown in Fig. 2. We can see that when the average mean-squared error was the smallest, lasso regression screened out five variables in Fig. 2a; at this time, λ = 0.09559. Among them, the selected variables were X1, X5, X8, X14, and X16. When the average mean-squared error was the smallest, lasso regression screened out seven variables in Fig. 2b; at this time, λ = 0.0822. Among them, the selected variables were X5, X6, X8, X10, X13, X14, and X16. The selected indicators were closely related to cumulative morbidity and mortality.

### Results of quantile regression

The results of quantile regression were different from the results of lasso regression, and quantile regression provided more comprehensive information. The specific content is presented in Table 1. The overall finding is that the mobility index, the ratio of safe drinking water, and the closure of non-essential businesses are related to the cumulative incidence. The closure of educational facilities, restrictions on gathering, and the closure of non-essential businesses are tied to cumulative mortality. In low- and medium-incidence countries, the mobility composite is connected with the cumulative incidence. In high-incidence countries, the closure of educational facilities and restrictions on gathering are linked to cumulative mortality.

**Table 1:** Results of the quantile regression

	Quantiles								
	0.1	0.2	0.3	0.4	0.5	0.6	0.7	0.8	0.9
*Y1*									
(Intercept)	− 3.88	− 1.35	− 14.06	− 8.92	− 11.81	− 27.89	− 25.56	− 27.45	− 3.62
X1	0.12	0.05	0.11	0.01	0.18	0.10	0.01	− 0.01	0.46
X5	0.04	0.07*	0.16*	0.17*	0.10*	0.31*	0.32*	0.35	0.31
X8	− 0.21	− 0.09	− 0.44	− 0.42*	− 0.52*	− 0.52	− 0.69	− 0.68	− 0.51
X14	− 2.73	− 3.36	− 4.67	− 5.39	− 4.26	2.12	− 0.39	− 0.20	− 28.64
X16	4.32	6.44*	5.12*	5.15*	8.30*	6.51*	8.81*	12.12	17.33
*Y2*									
(Intercept)	− 8.54	− 14.20	0.79	− 8.84	35.08	15.56	− 22.43	− 17.87	− 111.83
X5	0.08	0.01	− 0.04	0.09	0.19	0.23	0.54	0.74	1.44
X6	0.00	0.00	0.01	0.01	0.00	0.00	0.00	− 0.01	0.00
X8	− 0.30	− 0.69	− 1.09*	− 0.86	− 0.82*	− 1.28*	− 1.74*	− 1.47*	− 2.60*
X10	7.46	5.64	1.78	0.15	4.52	8.27	− 4.70	− 15.30	− 33.26
X13	3.83	− 7.50	− 57.16	− 51.27	− 48.17	− 44.13	− 31.18	− 20.35*	8.17
X14	− 6.59	7.77	39.84	41.69	− 10.86	− 6.85	1.25	− 14.87*	5.63
X16	3.87	10.08*	13.73*	12.66*	16.70*	21.59*	19.06*	25.98	1.94

*The coefficient is statistically significant

## Discussion

We found that the population prevention and control measures implemented by the government had an impact on the change in the Rt value. In most countries, the Rt value had a clear upwards trend in October. The most important factor in personal prevention and control is the mobility composite. Group prevention of the total testing, the closure of educational facilities, restrictions on large-scale gatherings, and commercial restrictions are very important for prevention and control. Moreover, the number of ICU beds and the average number of doctors in medical resources are key elements. Basic sanitation, such as the proportion of safe drinking water, has also had a certain impact on the COVID-19 epidemic.

The rate of masks used in individual prevention does not seem to be related to cumulative mortality or morbidity, which does not mean that the use of masks has no effect on prevention and control. Research on the effectiveness of masks for prevention and control has been confirmed [4]. The lasso regression identifies variables with a very large degree of correlation, so the mobility index generated by personal behaviour in prevention and control may be more important than wearing a mask. Second, one possible reason could be that the data on the rate of mask use come from social surveys, and there may be large errors.

Some studies have reached conclusions consistent with ours. The outcomes of the dynamic SEIR model show that the lockdown control measures implemented by China on 23 January 2020 were essential to ultimately reducing the scale of the COVID-19 epidemic [32]. Another study found that the early detection and isolation of cases prevented more infections than restrictions on travel and reduced contact. Our research also reveals a certain relationship between cumulative mortality and testing [33]. Some scholars have even proposed that the best strategy is to use both robot recognition and migration restriction strategies. European countries can also take this approach to reduce exposure to infection and provide help for the prevention and control of diseases [34]. Once the initial pandemic is under control, we must turn our attention to how to improve the adverse effects of the lockdown [35].

Studies have also indicated that medical resources are related to the mortality rate of COVID-19, which proves our research conclusions. Our study demonstrates that the number of doctors per capita and the number of hospital beds per capita are linked to the incidence or death of COVID-19 [36]. The experience in Wuhan implies that if medical resources become scarce, the government should establish temporary hospitals, and medical staff will be deployed from areas where the epidemic is relatively mild to ease the pressure in severely affected zones. Effective quarantine via quick detection prevents a larger outbreak [37, 38]. It is necessary to establish medically necessary, time-sensitive procedure scoring systems during the COVID-19 pandemic [39]. Within days or weeks, the health system is reorganised. We must optimise health resources. The fight against the disease occurs via a joint medical team composed of doctors, nurses, pharmacists, and respiratory therapists [40].

The ratio of safe drinking water in basic health resources has a relationship with the cumulative morbidity and mortality of COVID-19, which may be related to the fact that hand washing can reduce the number of hand viruses and achieve a certain prevention and control effect. Basic cleaning services are a prerequisite for infection prevention and control [41].

We did not include certain indicators—such as nurses per capita, pharmacists per capita, GDP, and GHS—in the regression model, which may be due to the relatively low degree of correlation. After the Ebola outbreak in 2014, the GHS index was developed to measure countries’ ability to respond to infectious disease outbreaks. Six core elements were evaluated: prevention, detection and reporting, response, the health system, compliance with norms, and the risk of infectious disease outbreaks. The higher the GHS score, the better the preparation.

The GHS index has low predictive value for the death outcomes of COVID-19, and we have reached the same conclusion [42, 43]. For example, the UK, which ranks second in terms of GHS index score, also bears a huge burden of disease [44]. However, the GHS index has a predictive effect on the burden of COVID-19, but in the opposite direction [45].

The outcomes of quantile regression indicated that low- and medium-incidence countries should pay more attention to personal prevention (mobility composite), and that high-incidence countries should close educational facilities and impose restrictions on gatherings.

Our research has some advantages. First, we described the prevalence and control of COVID-19 in European countries. Second, we included many independent variables to analyse their relationship with dependent variables. The independent variables mostly include individual and group prevention, medical resources, basic health facilities, and comprehensive indicators. Third, we used lasso regression to screen variables with a smaller error than traditional regression, and the results are more accurate. We further performed quantile regression to quantify the specific situation of each divided point, thus providing more information than traditional regression. Of course, our article also has some shortcomings. For example, Asmall number of proven effective prevention and control measures did not enter into our regression model, which may result from the accuracy of the data and the impact of the variables.

## Conclusions

We comprehensively described the status of COVID-19 prevention and control in European countries. We attempted to identify key factors in individual and group prevention measures, which can provide a policy basis for the prevention and control of epidemics in European countries.

## Data Availability

The datasets used and/or analysed during the current study are available from the corresponding author on reasonable request.
